# Extracellular circulating miRNAs as potential non-invasive biomarkers in non-small cell lung cancer patients

**DOI:** 10.3389/fonc.2023.1209299

**Published:** 2023-07-21

**Authors:** Justyna Raczkowska, Agnieszka Bielska, Adam Krętowski, Magdalena Niemira

**Affiliations:** ^1^ Clinical Research Centre, Medical University of Białystok, Białystok, Poland; ^2^ Department of Endocrinology, Diabetology and Internal Medicine, Medical University of Białystok, Białystok, Poland

**Keywords:** NSCLC, non-small cell lung cancer, miRNA, microRNA, biomarker, biofluids, serum, plasma

## Abstract

Non-small cell lung cancer (NSCLC) comprises 85% of all lung cancers and is a malignant condition resistant to advanced-stage treatment. Despite the advancement in detection and treatment techniques, the disease is taking a deadly toll worldwide, being the leading cause of cancer death every year. Current diagnostic methods do not ensure the detection of the disease at an early stage, nor can they predict the risk of its development. There is an urgent need to identify biomarkers that can help predict an individual’s risk of developing NSCLC, distinguish NSCLC subtype, allow monitor disease and treatment progression which can improve patient survival. Micro RNAs (miRNAs) represent the class of small and non-coding RNAs involved in gene expression regulation, influencing many biological processes such as proliferation, differentiation, and carcinogenesis. Research reports significant differences in miRNA profiles between healthy and neoplastic tissues in NSCLC. Its abundant presence in biofluids, such as serum, blood, urine, and saliva, makes them easily detectable and does not require invasive collection techniques. Many studies support miRNAs’ importance in detecting, predicting, and prognosis of NSCLC, indicating their utility as a promising biomarker. In this work, we reviewed up-to-date research focusing on biofluid miRNAs’ role as a diagnostic tool in NSCLC cases. We also discussed the limitations of applying miRNAs as biomarkers and highlighted future areas of interest.

## 1 Introduction

**Figure 1:**
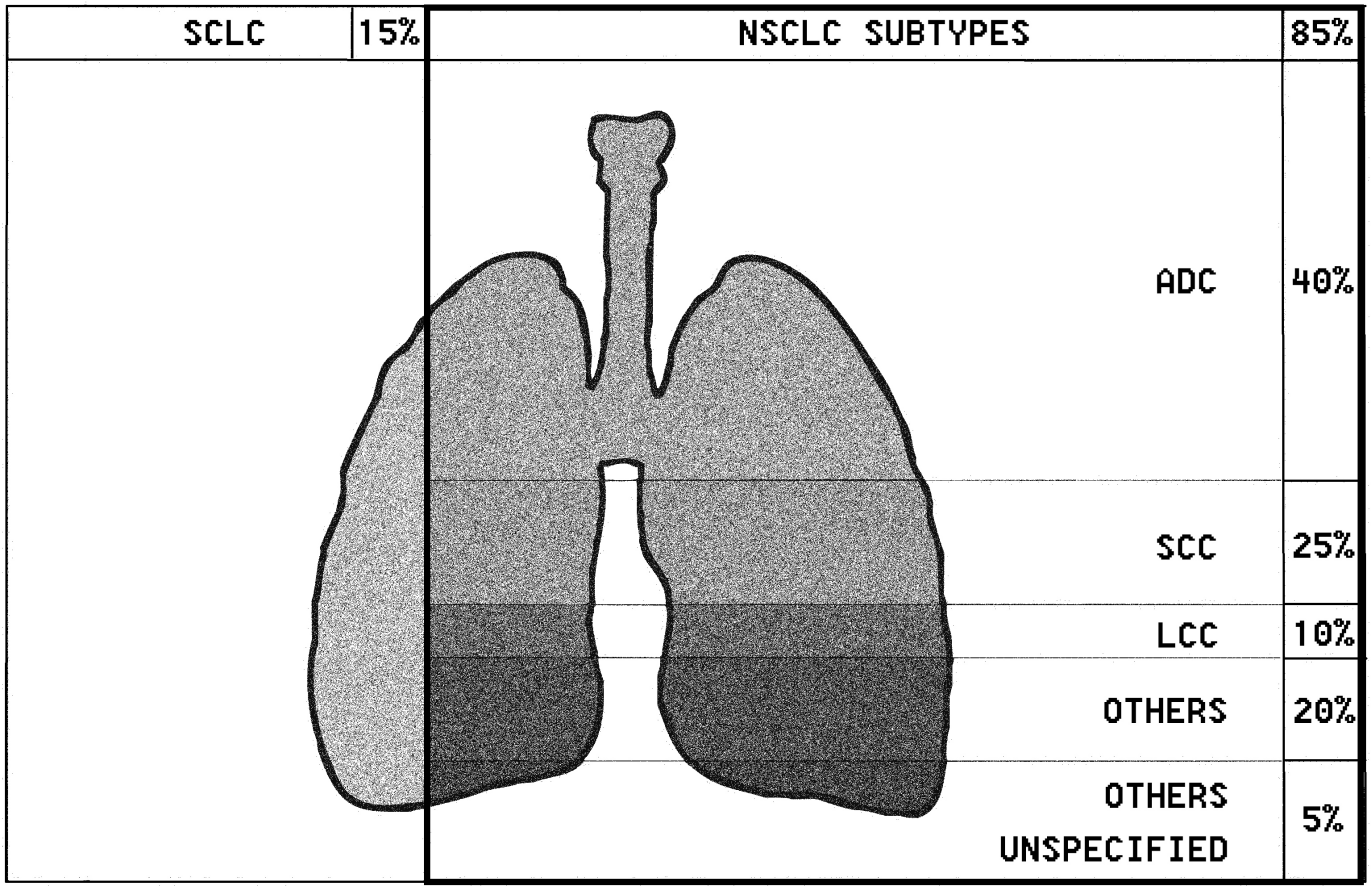
Lung cancer (lung carcinoma) is divided into two histologic types: non-small cell lung carcinoma (NSCLC) and small-cell lung carcinoma (SCLC). There are three main subtypes of NSCLC, including the following: adenocarcinoma (ADC), squamous-cell carcinoma (SCC), and large-cell carcinoma (LCC). Other subtypes include pulmonary enteric adenocarcinoma.

Lung cancer remains one of the most frequent and deadly cancers worldwide. The International Agency for Research on Cancer estimated approximately 2.2 million of new cases and 1.8 million deaths in 2020. Among males, lung cancer appears to be the first diagnosed cancer, whereas it ranks third for females. Lung cancer continues to be the leading cause of cancer death being overtaken only by breast cancer as the most commonly diagnosed one (1). Non-small cell lung cancer (NSCLC) is the first of two histologic lung cancer types, affecting approximately 85% of all cases. In this type, it is possible to distinguish three main subtypes: adenocarcinoma (ADC), relating to 40% of cases; squamous-cell carcinoma (SCC), which affects 25% of cases; and large-cell carcinoma (LCC), in 10% of cases (**Figure 1**) (2). The American Joint Committee on Cancer (AJCC) recommends using the TNM classification of lung cancer. This staging is established on the characteristics of the primary tumor (T), the degree of lymph node involvement (N) and metastasis status (M). Consequently, each patient is assigned a general stage (0, I, II, III, IV). The the most advanced stage is IV, while stages 0, I and II are considered to be early stages (3, 4).

The overall 5-year survival rate is 15% in NSCLC patients, which results from complex histology and genetics within subtypes of cancer, non-specific symptoms, and late diagnosis (5, 6). Unfortunately, lung cancer is often detected at late stages, when invasion and distant metastasis has already occurred. Nowadays, one of the initiating steps to diagnose lung cancer is a chest X-ray, which can expose a mass typical for cancer tumours. On that basis, more precise tests, such as bronchoscopy and liquid biopsy, can be performed to complete the diagnosis and establish the tumour subtype (7, 8). Currently, treatment is focused on surgery, chemotherapy, and radiotherapy, which is still insufficient to reduce NSCLC mortality (8). Molecular therapies are currently based mainly on tyrosine kinase inhibitors. Nevertheless, positive results can only be expected in NSCLC patients with *EGFR, BRAF* and *MET* mutations, as well as with rearrangement of the *ALK, ROS1* or *NTRK* genes. Likewise, immune checkpoint inhibitors (ICIs) provide a new and promising approach, but treatment is effective only in 20-30% of NSCLC cases. Despite the immunotherapy and recent development of targeted therapies, the World Health Organisation guidelines emphasized the importance of better subclassification of lung cancer (9). Accurate and detailed classification of subtype of NSCLC is often a challenge, due to limited diagnostic material or the need for additional techniques such as immunohistochemistry (10). New, accurate biomarkers could help distinguish the subtype of NSCLC at an early stage which would enable the implementation of early treatment. Moreover, a very important task is to identify biomarkers to predict treatment effects and response to therapies, which will allow the selection of personalised therapy (11).

**Figure 2:**
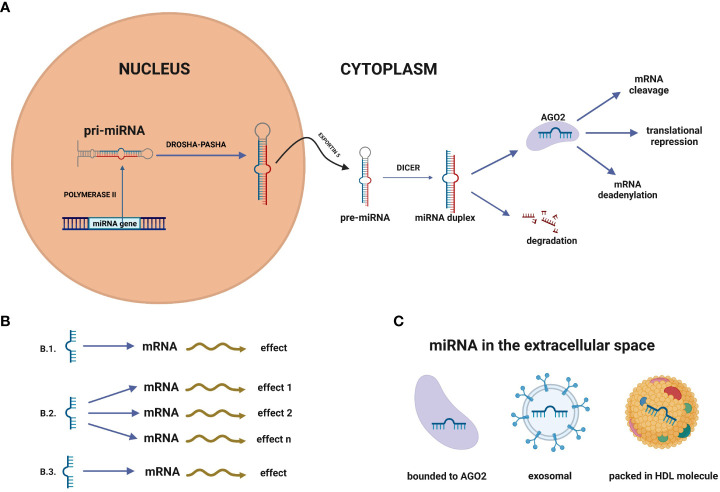
**(A)** MicroRNA biogenesis. MiRNA genes are transcribed in the nucleus by polymerase II to primary RNA (pri-miRNA). Pri-miRNA is transformed by the Drosha-Pasha complex into pre-miRNAs. In the next step, pre-miRNAs are transported to the cytoplasm by the nuclear transport protein – exportin 5. Subsequently, the enzyme Dicer converts pre-miRNA into double-stranded duplexes, which are loaded into the RNA Induced Silencing Complex (RISC). Only one miRNA strand is retained and bound to the RISC component - protein argonaute (AGO2) as mature miRNA, while the second is usually degraded. **(B)** MiRNA networks. **(B.1)** Single miRNA-mRNA interplay, **(B.2)** One miRNA can affect many miRNAs, **(B.3)** Single miRNA can impact different miRNAs to gain the biological effect. **(C)** In the extracellular space, miRNAs can be packed in vehicles (exosomes, microvesicles, apoptotic bodies) but also into high-density lipoprotein (HDL) molecules or bound to proteins, such as Ago2.

MicroRNAs (miRNAs) appear to be one of the most abundant RNA in the cells, making them promising molecules for detecting NSCLC and other cancers. MiRNAs are a group of short (about 21-23 nucleotide-long) and single-stranded particles that function through negative post-transcriptional regulation of gene expression. MiRNAs genes are primarily located in the inter-gene regions. They can exist as independent transcription units between protein-coding sequences. About 25% of the miRNA coding sequences in humans are located in introns. MiRNA genes are also found in exons and can form polycistronic clusters having identical regulatory sequences (12–14). The first stage of miRNAs’ canonical formation occurs in the cell nucleus, where the miRNAs are transcribed into pri-miRNAs (**Figure 2**). The primary transcripts, which can be longer than 1000 nucleotides, include an approximately 70-nucleotide double-stranded fragment in the form of a “hairpin”. This fragment is recognised and transformed by the Drosha-Pasha complex into pre-miRNAs, having a phosphate group at the 5’ end and two unpaired nucleotides at the 3’end. The obtained pre-miRNAs are then transported to the cytoplasm by the nuclear transport protein - exportin 5. Subsequently, the enzyme Dicer converts pre-miRNA into double-stranded duplexes (dsRNAs), which are loaded into the RNA Induced Silencing Complex (RISC). Only one miRNA strand is retained and bound to the RISC component - protein argonaut (AGO2) as mature miRNA, while the second is usually degraded. The target mRNA sequences are recognised by mature miRNAs mainly in the 3’ untranslated region (3’ UTR), although recognition is sometimes found in the 5 ‘untranslated region (5’UTR). Consequently, these targeted mRNAs are silenced by cleavage of the miRNA, translational repression, mRNA deadenylation, or a combination of these processes (15–17).

It is observed that miRNAs are tissue-specific, and their concentration changes are seen in affected and healthy tissues (18–21). The multifunctionality and multidirectional activity of miRNA molecules underline their essential role in organism functioning. They play an important role in many processes, such as proliferation, differentiation, gene silencing, carcinogenesis, apoptosis, and cell survival (22–25). Furthermore, it has been found that specific miRNAs are associated with metastasis, inter alia, through the regulation of epithelial-mesenchymal transition (26).

A solid tumour releases miRNAs into the body fluids such as blood, serum, plasma, amniotic fluid, saliva, urine and peritoneal fluid (22, 23, 27). In the extracellular space, miRNAs can be packed in vehicles (exosomes, microvesicles, apoptotic bodies) but also into high-density lipoprotein (HDL) molecules (24) or bound to proteins, such as Ago2 (**Figure 2B**) (28, 29). It has been found that circulating miRNAs are protected from endogenous RNase activity (23), and have a low susceptibility to extreme temperatures and pH changes (30). All these features make them stable even after repeated freezing and thawing cycles of diagnostic material. Collecting biofluids from patients by liquid biopsy is minimally invasive and easy; thus, diagnostics could be more accessible and less expensive (31). MiRNAs can act as oncogenes (oncomiRs) by negatively regulating tumor suppressor genes involved in apoptosis and differentiation. Some miRNAs act as tumor suppressors, so under-expression of the gene leads to cancer development (32). MiRNA can act as an oncomiR and suppressor in specific conditions. For example, miR-196 was examined to be up-regulated in the oesophagus, glioblastoma and colon cancer, but other studies have verified it to act as a tumour suppressor (33).

MiRNAs can act as oncogenes (oncomiRs) by negatively regulating tumor suppressor genes involved in apoptosis and differentiation. Some miRNAs act as tumor suppressors, so under-expression of the gene leads to cancer development (32).

Many bioinformatic tools for analysis require a good understanding of biological processes. The increasing use of molecular techniques has allowed researchers to identify miRNAs and expand the knowledge about the targets of these molecules. The most commonly used process for expression analysis of miRNA is quantitative reverse transcriptase polymerase chain reaction (qPCR/RT-qPCR) which is a gold standard. Other methods widely used include northern blotting, next-generation RNA sequencing, and microarrays (11). One of the most advanced methods of detecting miRNAs is the nCounter platform (Nanostring Technology). This method offers direct RNA detection without an amplification process, so it is based on direct multiplexed measurement of gene expression (34). The freely available database miRBase (http://www.mirbase.org) is constantly enriched in information about miRNAs. It provides knowledge about mature and hairpin miRNA sequences, their location and biogenesis precursors. There are also links to literature references, annotations and databases that contain predicted and experimentally validated targets of miRNAs (35). Discovering the miRNA sequences provides bioinformatics tools to predict its targets. It is possible due to the recognition of mRNA matches to miRNA seed regions which are 6-8 pairs long. Statistics methods have revealed that a single miRNA could have nearly hundred targets, which may result in a sum of effects determining a typical phenotype (36). In addition, miRNA networks show that one miRNA can impact different miRNAs to act synchronically (**Figure 2C**) (37). Clusters of two or more miRNAs are commonly related to several cell functions. For example, the miR-17-92 cluster is connected with the development of the lungs, immune system, and cancer development (38).

Many studies investigating miRNA activity in cancer have focused on its potential as diagnostic biomarkers, predictors of prognosis and drug efficiency. It could also effectively classify cancer subtypes (39) and predict metastatic outcomes (40). Research evidence indicates the role of biofluid miRNAs in many cancers, such as ovarian cancer (41), prostate cancer (42), liver cancer (43), and NSCLC (30). Lin et al. (44), as early as 2010, published a review focusing on the significant role of miRNAs in lung cancer. It is well known that miR-21 is involved in many cancer processes, such as tumorigenesis, progression and metastasis (45, 46). Numerous studies demonstrated that miR-21 is up-regulated in hepatocellular carcinoma (47), gastric cancer (48), prostate cancer (49), and also in lung cancer (45). It was shown that this miRNA is highly expressed in NSCLC patients, and inhibition of miR-21 reduces the proliferation, migration and invasion of cancer cells (45). Yu et al. (50) indicated that miR-10a is up-regulated in NSCLC patients’ tissue. Furthermore, authors showed that this miRNA might promote the progression of cancer cells by targeting *PTEN/AKT/ERK* signalling pathway. Interestingly, in the previous study by Markou et al. (51), plasma miR-10a was described as a promising biomarker for NSCLC. It has been proven that miRNA-200a-3p showed under-expression in the NSCLC tumour tissues and A549 cell lines. Insulin receptor substrate 2 (*IRS2*) was shown to be a direct target for this miRNA (52).

This review focuses on recent advances in the role of biofluid-circulating miRNAs in NSCLC. We organize and present here the latest reports, which could support the search for new diagnostic, prognostic and predictive biomarkers for NSCLC. This manuscript includes recently studied miRNAs from exosomes (with “exosomal” prefix in the text, with “ex” in tables), protein-bounded miRNAs (without the prefix in tables) and vehicle-derived (“sEVs”) miRNAs.

The paragraphs of this manuscript are divided according to the potential application of the studied miRNAs. Notably, the results of many studies point to more than one possible use for miRNAs, so the results of one paper are present in more than one paragraph. However, in the tables, we decided to list the latest reports concerning miRNAs in chronological order: serum (**Table 1**), plasma (**Table 2**), and sputum (**Table 3**), to avoid repetition in the tables. The most promising miRNAs for the diagnosis of NSCLC are presented in **Table 4**.

**Table 1:** Latest reports concerning miRNAs in serum of NSCLC patients.

miRNA	Expression in Research Group vs Controls	Number of cases	Significant Findings	Methods	Application in NSCLC	Year of Publication	Reference
miR-30	down	108 NSCLC/108 controls	levels of this miRNA correlated with lymph node metastasis, tumour size, TNM stage, and differentiation degree	qRT-PCR	diagnosis, prognosis	2020	(53)
exosomal miR-1246	up	105 NSCLC/50 NMLD patients/50 controls	miR-1246 may help distinguish NSCLC patients from controls and NMRD-diseased patients	qRT-PCR	diagnosis, prognosis	2020	(54)
exosomal miR-620	down	235 NSCLC/231 controls	miR-620 was associated with alcohol consumption, distant metastasis and chemotherapeutic effect	TEM, qNano, and western blot; validation: qPCR	diagnosis, prediction	2020	(55)
miR-762	up	148 NSCLC/60 controls	high levels of miR-762 were associated with histological grade, clinical stage and lymph node metastasis	qRT-PCR	diagnosis, prognosis	2020	(56)
miR-519d	down	130 NSCLC/50 controls	miR-519d under-expression was associated with lymph node metastases, clinical stage, and a poor prognosis	qRT-PCR	diagnosis, prognosis	2020	(57)
ex-miR-5684, ex-miR-125b-5p	down	330 NSCLC/312 controls	levels of that miRNAs were associated with metastasis (P < 0.0001), chemotherapeutic effect (P = 0.007)	TEM, qNano, western blot, microarrays, qPCR	diagnosis, prediction	2020	(58)
miR-4687-3p	up	training cohort: 20 NSCLC/20 controls, validation cohort: 30 NSCLC/30 controls	this miRNA plays an important role in NSCLC development	microarray, qRT-PCR	diagnosis	2020	(59)
miR-145, miR-20a	down: miR-145, up: miR-20a	32 NSCLC/33 other lung cases/23 controls	miR-145 and miR-20a may be NSCLC biomarkers	qRT-PCR	diagnosis	2020	(60)
miR-942, miR-601	up	125 NSCLC/40 benign lung diseases/60 controls	these miRNAs performed better than CEA, CYFRA21-1, and the SCC antigen in the early detection of NSCLC	qRT-PCR	diagnosis, prognosis	2020	(61)
miR-15a-5p, miR-320a, miR-25-3p, miR-192-5p, miR-let-7d-5p, miR-let-7e-5p, miR-148a-3p, miR-92a-3p	down	75 NSCLC/40 controls	miR-320a, miR-25-3p, miR-192-5p, miR-let-7d-5p, miR-148a-3p, and miR-let-7e-5p were significantly correlated with gender of patients	small RNA seq., validation: qPCR	diagnosis	2020	(62)
miR-375, miR-10b-5p	down only in SCC	75 NSCLC/40 controls	miR-375 levels were significantly correlated with lymph node involvement, while both miRNAs with pleural effusion	small RNA seq., validation: qPCR	prognosis	2020	(63)
ex-miR-20b-5p, ex-miR-3187-5p	down	276 NSCLC/282 controls	integrating those miRNAs could distinguish the patients at an early stage from healthy controls	qNano, TEM, western blot, qRT-PCR, microarrays	early-stage diagnosis	2020	(64)
miR-17-92 gene cluster: miR-17, miR-18a, miR-19a, miR-19b-1, miR-20a and miR-92a	down: miR-17 (stage II, III), miR-18a (stage II), miR-19a/b-1 (stage II, III), miR-20a (stage I, II, III), miR-92a-1 (stage II, III); up in stage IV: miR-17, miR-18a, miR-20a, miR-92a-1	74 NSCLC/23 controls	miR-17-92 gene cluster might be helpful as a diagnostic marker in NSCLC	qRT-PCR	diagnosis	2020	(65)
miR−518b	up	118 NSCLC/60 controls	higher miR-518b expression was associated with larger tumour size, lymph node metastasis, advanced TNM stage, and with poor overall survival in NSCLC patients	qRT-PCR	diagnosis, prognosis,	2020	(66)
ex-miR-378	up	103 NSCLC/32 NMLD patients/60 controls	miR-378 could be potentially used as an indicator of radiotherapeutic response in NSCLC	qRT-PCR	diagnosis, prediction	2020	(67)
miR-185	down	146 NSCLC/50 carcinoma in situ/25 NMLD/80 controls	serum miR-185 expression was progressively decreased in healthy controls, patients with NMLD, carcinoma *in situ* and NSCLC	qRT-PCR	early detection, prognosis	2020	(68)
miR-181a-5p, miR-1228-3p	down: miR-181a-5p, up: miR-1228-3p,	50 NSCLC/30 controls	miR-1228-3p and miR-181a-5p levels were significantly associated with OS	qRT-PCR	diagnosis, prognosis	2020	(69)
miR-3195,	down	75 NSCLC/40 controls	miR-3195 as an independent prognostic factor for OS	qRT-PCR	prognosis	2020	(70)
miR-1249-3p,	down	75 NSCLC/40 controls	miR-1249-3p might predict therapeutic response to chemotherapy	qRT-PCR	therapeutic response	2020	(70)
ex-miR-1269a, ex-miR-9-3p, ex-miR-205-5p and ex-miR-210-5p	down	147 NSCLC/139 controls	panel of four tested exosomal miRNAs may serve as noninvasive diagnostic biomarker for NSCLC	qRT-PCR	diagnosis	2020	(71)
miR-186	down	62 NSCLC/60 controls	miR-186 and IL-1β might serve as potential biomarkers for NSCLC	qRT-PCR	diagnosis	2020	(72)
miR−130a, miR−25 and miR−191	up	84 NSCLC/42 controls	these miRNAs may have an oncogenic role in the radiation−mediated metastasis of NSCLC	qRT-PCR	prediction	2020	(73)
ex-miR-21/miR-let-7a ratio	up	75 NSCLC/23 benign pulmonary nodules/18 pulmonary inflammation diseases/24 controls	this ratio can be used to distinguish NSCLC from benign pulmonary disease patients	qRT-PCR	diagnosis	2020	(74)
ex-let-7e	down	105 NSCLC/50 controls	higher levels of that miRNA had an anticarcinogenic properties against NSCLC	TEM, cell culture, qRT-PCR, western blot	prognosis	2020	(75)
miR-21-5p, miR-141-3p, miR-222-3p, and miR-486-5p, ex-miR-146a-5p, ex-miR-486-5p	up	48 NSCLC/32 benign lung lesion/48 controls	these miRNAs could further improve early diagnosis of NSCLC	TEM, Western blot, qRT-PCR	early diagnosis	2020	(76)
miR-184, miR-191	down: miR-184, up: miR-191	100 NSCLC/59 pneumonia/51 controls	these miRNAs might be used as biomarkers to diagnose and predict the curative effect of treatment in patients with NSCLC	qRT-PCR	diagnosis, prediction	2020	(77)
panel of: miR-let-7a-5p, miR-375, miR-1-3p, miR-1291, and miR-214-3p	down: miR-let-7a-5p and miR-375; up: miR-1-3p, miR-1291, miR-214-3p	744 NSCLC/944 controls	the five-miRNA panel could detect early-stage (I and II) NSCLC	qRT-PCR	prognosis	2020	(78)
miR-93-5p, miR-18a	up	107 NSCLC/42 controls	combination of these miRNAs had a high diagnostic value	qPCR	diagnosis, prognosis	2021	(79)
ex-miR-382	down	126 NSCLC/60 controls	level of this miRNA was increased after surgery in most of the patients	qRT-PCR	prognosis	2021	(80)
miR-891a-5p	up	120 NSCLC/68 controls	this miRNA had a high diagnostic value (AUC = 0.904) in NSCLC patients	cell cultures, qRT-PCR	diagnosis, prognosis,	2021	(81)
miR-23a, miR-let7i	down	31 NSCLC/21 controls	miR-23a and miR-let7i might serve as non-invasive markers in NSCLC	qPCR	diagnosis	2021	(82)
ex-miR-7	up	35 NSCLC/35 controls	up-regulation of this miRNA was associated with longer survival of patients and with a solid reaction to gefitinib treatment	cell cultures, western blot, qRT-PCR	prediction	2021	(83)
miR-629	up	166 NSCLC/70 nonmalignant lung diseases/100 controls	miR-629 had better performance for distinguishing NSCLC patients from healthy ones than CYFRA 21-1 and CEA	qRT-PCR	prognosis	2021	(84)
ex-miR-192 and ex-miR-194	down	100 NSCLC/100 controls	lower levels of miR-192 and miR-194 were associated with NSCLC TNM stage, metastases and histopathological grade	qRT-PCR	prognosis	2021	(85)
ex-miR-184, ex-miR-3913-5p	up (after the onset of osimertinib resistance)	67 NSCLC/10 controls	these miRNAs may serve as biomarkers to indicate osimertinib resistance	cell cultures, TEM, RNA−seq., qRT-PCR,	prediction	2021	(86)
ex-miR-1169, ex-miR-260	up	64 NSCLC/20 controls	those miRNAs could be biomarkers to distinguish wild-type EGFR and mutant EGFR NSCLC patients in early-stage	TEM, cell cultures, western blot, small RNA seq., validation: qRT- PCR	prediction	2021	(87)
miR-205-5p	up	75 NSCLC/62 controls	increased level of serum miR-205-5p was associated with patients’ gender, clinical stage, and drinking status	qRT-PCR	diagnosis, prognosis	2021	(88)
ex-miR-433	down	33 NSCLC/19 controls	this miRNA might help distinguish chemotherapy sensitive patients from resistant ones	TEM, western blot, qRT-PCR	prediction	2021	(89)
miR-138-5p	down	18 NSCLC/18 controls	ROC analysis revealed that miR-138-5p might be a promising diagnostic biomarker	qRT-PCR	diagnosis	2022	(90)
miR-31-3p	up	140 NSCLC (metastatic NSCLC group – 58; non-metastatic group – 82)/60 controls	higher miR-31-3p levels, TNM classification, and lymphatic metastasis might be used as risk factors and independent predictors of bone metastasis	qRT-PCR	prognosis	2022	(91)
miR-30a-5p	down	20 ADC/20 controls	this miRNA correlated with progression and immune infiltration	qRT-PCR	prognosis	2022	(92)

ADC, adenocarcinoma; JCC, American Joint Committee on Cancer; CEA, carcinoembryonic antigen; CYFRA21-1, cytokeratin 19 fragments; EGFR, epidermal growth factor receptor; ex-, exosomal; HOXA5, homeobox A5; IL-1b, interleukin-1b; NSCLC, non-small cell lung cancer; NMLD, non-malignant lung diseases; OS, overall survival; qNano, TRPS (Tunable Resistive Pulse Sensing) particle analyzer; qPCR/qRT-PCR, quantitative polymerase chain reaction/real-time quantitative reverse transcription PCR; SCC, squamous-cell carcinoma; small RNA seq., small RNA sequencing; TEM, transmission electron microscope; TNM, tumour node metastasis; ZEB2, zinc finger E-box binding homebox 2.

**Table 2:** Latest reports concerning miRNAs in plasma of NSCLC patients.

miRNA	Expression in Research Group vs. Controls	Number of cases	Significant Findings	Methods	Application in NSCLC	Year of Publication	Reference
miR‐590‐5p	down	80 NSCLC/80 controls	negative correlation of that miRNA was observed in NSCLC prognosis	qRT-PCR	prognosis	2020	(93)
ex-miR-1273a	down	49 NSCLC/Cel-miR-39	miR-1273a level was correlated with worse therapeutic outcomes in advanced NSCLC patients receiving platinum-based chemotherapy	TEM, western blot, qRT-PCR	prediction	2020	(94)
ex-miR-320b, ex-miR-320c, ex-miR-320d, ex-miR-125b-5p	down: miR-125b-5p; up: miR-320b, miR-320c, miR-320d	30 NSCLC/7 controls	patients with down-regulated miR-125b-5p might have greater T-cell function and respond well to immunotherapy	TEM, small RNA seq	prediction	2020	(95)
miR-21, miR-128, miR-155, miR-181a	up	128 NSCLC/19 controls	miR-128 and miR-155 could be independent predictors in platinum-based chemotherapy: of worse OS (miR-128) and worse OS in SCC (miR-155)	qRT-PCR	prediction	2020	(96)
miR-1247-5p, miR-301b-3p, miR-105-5p	up	154 NSCLC/146 controls	these miRNAs can serve as potential NSCLC early diagnosis biomarkers	microarrays, validation: qPCR	early diagnosis	2021	(97)
miR-21	up (in patients who achieved a partial/complete treatment response)	39 NSCLC	this miRNA might be used to monitor NSCLC patients during the EGFR-TKIs treatment	qRT-PCR	prediction	2021	(98)
ex-miR-1246, ex-miR-96	up	52 NSCLC (27 radioresistant NSCLC, 25 radiosensitive NSCLC)/45 healthy controls	These miRNAs may serve as NSCLC biomarkers	qRT-PCR	diagnosis	2021	(99)
ex-miR-96	up	52 NSCLC (27 radioresistant NSCLC, 25 radiosensitive NSCLC)/45 healthy controls	exosomal miR-96 could be a biomarker of radioresistant NSCLC	TEM, western blot, qRT-PCR	prediction	2021	(99)
miR-183, miR-210, miR-182, miR-144	down:miR-144 (in HPV-DNA-positive NSCLC subjects); up: miR-183, miR-210, miR-182,	100 NSCLC/52 controls	these miRNAs might serve as biomarkers to identify circulating HPV-DNA-positive NSCLC	qRT-PCR	diagnosis of HPV-DNA-positive NSCLC	2021	(100)
miR-216b	down	80 NSCLC/40 controls	miRNA-216b levels were associated with tumour staging and negatively correlated with 18F-FDG uptake in NSCLC	qRT-PCR	diagnosis, prediction	2021	(101)
miR-320a	down	80 NSCLC/80 controls	levels of that miRNA were associated with tumour size, TNM stage and lymph node metastasis	qRT-PCR	prognosis	2021	(102)
miR-210, miR-1290, miR-150,miR-21-5p	up	training set: 40 NSCLC/20 controls, validation: 88 NSCLC/50 benign lung disease/40 controls	up-regulation of miR-210 and miR-150 were associated with worse disease−free survival time in NSCLC patients	qRT-PCR	diagnosis, prognosis (only miR-210 and miR-150)	2021	(103)
ex-miR-574-5p, ex-miR-328-3p, ex-miR-423-3p	down: miR-574-5p, up: miR-328-3p, miR-423-3p	30 *EGFR/ALK genes* positive NSCLC/14 controls	these miRNAs may serve as predictors of bone metastasis in NSCLC patients	small RNA seq.	prognosis	2021	(104)
miR-202, miR-26a	up	125 NSCLC/33 controls	miR-202 may be an independent prognostic factor for shorter progression-free and OS	qRT-PCR	prognosis of treatment outcomes	2021	(105)
ex-miR-1260b	up	48 NSCLC/48 controls	miR-1260b up-regulation was associated with high-grade disease, metastasis, and poor survival	TEM, western blot, qRT-PCR	prognosis	2021	(106)
miR-200c, miR-34a	down: miR-34a, up: miR-200c	69 NSCLC/33 controls	levels of these miRNas were associated with the response and outcome in advanced NSCLC patients treated with anti-PD1 immunotherapy	qRT-PCR	prediction	2022	(107)
sEVs-miR-152-3p, sEVs-miRNA-1277-5p	up	10 NSCLC/10 SCLC/15 controls	sEVs plasma miRNA-152-3p and miRNA-1277-5p might be used to diagnose early-stage NSCLC patients	TEM, western blot, small RNA seq., validation: qRT-PCR	diagnosis	2022	(108)
miR-340, miR-450b-5p	down: miR-340, up: miR-450b-5p	120 NSCLC/120 controls	these miRNAs might be independent biomarkers of survival in non-metastatic NSCLC	qRT-PCR	diagnosis, prognosis	2022	(109)
miR-22-3p, EV-let-7b-5p, EV-miR-184	down: EV-miR-22-3p, EV-miR-184; up: EV-let-7b-5p	20 NSCLC/6 false-positive NSCLC/14 non-cancer lung nodules	these miRNAs could distinguish NSCLC patients from high-risk subjects	small RNA seq., western blot, qRT-PCR	diagnosis	2022	(110)
miR-34c-5p	down	19 NSCLC males/34 males controls	this miRNA could potentially be a diagnostic NSCLC biomarker	qRT-PCR	diagnosis	2022	(111)
ex-miR-130a-3p	up (in relapsed patients compared to non-relapsed)	67 non-metastatic NSCLC/30 metastatic NSCLC	combining of miR-130a-3p and fibrinopeptide A expression was significantly associated with disease-free survival	microarrays, droplet digital PCR, qPCR, nCounter platform, cell cultures	prediction	2022	(112)
EV-miR-625-5p	down	22 SCC/45 ADC	levels of that miRNA could distinguish patients with longer survival	nCounter platform	prediction of survival and response	2022	(113)

18F-FDG, 18F-Fluorodeoxyglucose; EGFR-TKIs, epidermal growth factor receptor tyrosine kinase inhibitors; EV, extracellular vesicle; ex-, exosomal; HPV, human papilloma virus; NSCLC, non-small cell lung cancer; OS, overall survival; RNA seq., RNA sequencing; sEVs, small extracellular vesicles; STAT3, signal transducer and activator of transcription 3; TEM, transmission electron microscope.

**Table 3:** Latest reports concerning miRNAs in sputum of NSCLC patients.

miRNA	Expression in Research Group vs. Controls	Number of cases	Significant Findings		Methods	Application in NSCLC	Year of Publication	Reference
miR-21, miR-32, miR-210	up	training set: 46 NSCLC/55 controls; testing set: 57 NSCLC/62 controls		these miRNAs and two sputum small nucleolar RNAs (SNORD66, SNORD78) had a synergistic effect for lung cancer detection	qRT-PCR	early diagnosis	2016	(114)
miR-31, miR-210	up	discovery cohort: 68 NSCLC/66 cancer-free smokers, validation cohort: 49 NSCLC/50 cancer-free smokers		combination of these two miRNAs with 1 PBMC miRNA (miR-19b-3p) had higher diagnostic value than the individual panel	qRT-PCR	early diagnosis	2018	(115)
miR‐145, miR‐126, miR‐7	down	30 NSCLC/30 controls		these miRNA could be potential biomarker for NSCLC due to their high AUC value and stability in the sputum	qRT-PCR	diagnosis	2018	(116)
miR-31-5p, miR-210-3p	up	76 NSCLC/72 cancer-free smokers		combining these miRNAs and miR‐21‐5p from plasma had the highest diagnostic value	qRT-PCR	early detection	2019	(117)
miR-21, miR-31, miR-210, miR-486	down: miR-486; up: miR-21, miR-31, miR-210	32 NSCLC/33 cancer-free smokers		these miRNAs could potentially be NSCLC biomarkers	small RNA seq., validation: qRT-PCR	diagnosis, therapy	2020	(118)
miR-31-5p, miR-210-3p	up	40 NSCLC/36 cancer-free smokers		combination of these miRNAs with sputum DNA methylation RASSF1A, plasma miR-21-5p and miR-126 had the highest diagnostic value	droplet digital PCR	diagnosis	2021	(119)

NSCLC, non-small cell lung cancer; PBMC, peripheral blood mononuclear cells; qPCR/qRT-PCR, quantitative polymerase chain reaction/real-time quantitative reverse transcription PCR; RASSF1A, ras association domain family member 1; small RNA seq., RNA sequencing.

**Table 4:** The most promising miRNAs in NSCLC diagnosis.

miRNA/panel	Source of miRNA	AUC	Sensitivity (%)		Specificity (%)	Application in NSCLC	Year of Publication	Reference
miR-762	serum	0.92		81.8	93.33	distinguishing NSCLC patients at the clinical stage I from healthy controls	2020	(56)
let-7d-5p	serum	0.91		76	100	distinguishing NSCLC patients from healthy controls	2020	(62)
miR-17 + miR-20a	serum	0.94		98	0.90	2-miRNA panel for stage I-III NSCLC diagnosis	2020	(65)
miR-518b	serum	0.91		88.1	81.7	distinguishing NSCLC patients from healthy controls	2020	(66)
miR-185 + CEA	serum	0.93		97.3	78.7	distinguishing patients with carcinoma from healthy controls	2020	(68)
ex-miR-1228-3p	serum	0.91		0.77	0.89	distinguishing NSCLC patients from healthy controls	2020	(71)
miR-21-5p, miR-141-3p, miR-222-3p, miR-486-5p) and two exosomal ones (miR-146a-5p and miR-486-5p	serum	0.96		85.42	92.5	early diagnosis of NSCLC patients	2020	(76)
miR-184 + miR-191	serum	0.929		89	84.75	distinguishing NSCLC patients from pneumonia subjects	2020	(77)
miR-184 + miR-191	serum	0.925		87	86.25	distinguishing NSCLC patients from pneumonia subjects	2020	(77)
miR-891a-5p	serum	0.904		82.5	80.9	distinguishing the patients with NSCLC from the healthy controls	2021	(81)
miR-210 + miR-144	plasma	0.98		93.7	98	detecting HPV-DNA-positive NSCLC patients	2021	(100)
panel of: miR-210, miR-1290, miR-150 and miR-21-5p	plasma	0.98		93.75	98	differentiating patients with stage II-IIIA NSCLC from healthy individuals	2021	(103)
miR-31 and miR-210 and miR-19b-3p	sputum, miR-19b-3p (from PBMC)	0.92		82.3	87.9	distinguishing the patients with NSCLC from the healthy controls	2021	(120)

MiRNAs with high AUC>0.9, with high specificity or/and specificity (>80%).

We searched the Pubmed database with keywords: miRNA, NSCLC, biomarkers AND serum/plasma/sputum/saliva/urine to find out recent research studies. In the case of serum and plasma, we selected research studies from 2020 to 2023. Due to the small amount of research on sputum, we selected research studies from 2016 to 2022. Moreover, we excluded studies from journals with an Impact Factor below 2.0 and those with the small study groups (<10).

## 2 Circulating miRNAs as diagnostic biomarkers for NSCLC

The high mortality rate from NSCLC is related to diagnosing patients in advanced stages. Early detection might improve patient outcomes and prevent complications. Diagnostic biomarkers are needed to determine whether a patient has NSCLC, discriminate it from other disease entities, and help determine its exact subtype of NSCLC. The importance of diagnostic markers is invaluable, as their results can be potentially used for later predicting the disease’s progression and the patient’s treatment response. Several studies have been carried out that have indicated the diagnostic potential of miRNAs in NSCLC.

In 2020, Yu (53) and Sui indicated that serum miR-30 might be a diagnostic biomarker of NSCLC patients. Levels of serum and tissue miR-30 were observed as significantly decreased compared to healthy controls. A receiver operating characteristic curve (ROC) analysis showed a high diagnostic accuracy with an area under the curve (AUC) value was 0.802, with 75.9% sensitivity and 76.0% specificity. It is worth conducting more extensive research on larger cohorts of NSCLC patients since miR-30 family members are proven to be involved in oncogenesis and invasion in different types of tumours (121). One of the recent studies focused on the clinical significance of serum exosomal miR-1246 in NSCLC. The authors showed for the first time a significant up-regulation of that miRNA in NSCLC patients than in non-malignant respiratory disease patients and healthy controls. Moreover, ROC analysis revealed that this miRNA differentiated early-stage NSCLC patients from healthy and non-malignant respiratory disease patients with an AUC value of 0.827 and 0.757, respectively (54). According to the research of Tang et al. (55), exosomal-derived serum miR-620 may be another promising diagnostic biomarker of NSCLC. Microarrays and subsequent validation via qPCR were performed using serum exosomes from 235 NSCLC patients and 231 healthy donors. The under-expression of miR-620 levels has been observed in NSCLC and early-stage patients’ exosomes compared to healthy individuals. The strengths of this study were the validation performed and the size of the study group. In addition, the study and control groups were characterized by the absence of any immunological, endocrine or metabolic diseases in the study and control groups. The results of another study by Chen et al. (56), showed up-regulation of serum miR-762 in NSCLC patients with the advanced clinical stage (III-IV), positive lymph node metastasis and poorly differentiated tumors towards patients at the early clinical stage. The combination of carcinoembryonic antigen (CEA), and cytokeratin 19 fragments (CYFRA21-1) with serum miR-762 enhanced the diagnostic efficiency for NSCLC. It was the first research to demonstrate that the level of serum miR-762 was significantly up-regulated in NSCLC patients, but validation on larger cohorts is needed to confirm those reports. It has been shown that miR-519b in NSCLC tissue and serum samples was markedly lower. ROC analysis demonstrated that serum miR-519d levels could discriminate NSCLC patients from healthy controls with an AUC of 0.855 (p < 0.0001). When the cut-off value was 0.22, the sensitivity and specificity were 98.1% and 91.8% (57). Altered expression of miR-519d was previously observed in other cancers (122, 123), so more accurate high-throughput cohort studies are needed to assess the diagnostic value of miR-519 for NSCLC. The study of Zhang et al. (58) has shed light on the potential utility of miR-5684 and miR-125b-5p in NSCLC diagnosing. Firstly, the authors used transmission electron microscopy (TEM), particle analyzer (qNano) and western blots to characterize the exosomes, then screened them out by microarrays to finally be verified by qPCR. The highest diagnostic accuracy (AUC = 0.863) of tested miRNAs was reached after combining it with two tested markers – 19 CYFRA21-1 and CEA. Interestingly, miR-5684 expression was markedly lower in patients in the T1-T4 compared to healthy subjects, whilst miR-125-5p was lower at T2 and higher stages. Moreover, miR-125b-5p allowed for the precise distinction of patients with the early stage (I and III) and advanced stage (III and IV). Another research was conducted using microarrays to screen 2.549 miRNAs in serum samples of NSCLC patients. Subsequently, qRT-PCR validation helped select miR-4687-3p, which next showed the highest diagnostic accuracy of NSCLC from the other five miRNAs (miR-1915-5p, miR-432-3p, miR-4488, miR-520a-5p, and miR-6087). On that basis, a comparison with information from The Cancer Genome Atlas (TCGA) database was made, what had confirmed that the level of miR-4687-3p was significantly higher in NSCLC tissues than in normal lung tissues (*p* < 0.05) (59). There is a need to identify the underlying molecular mechanisms of miR-484, therefore the authors plan to implement it in their further research. Some studies have focused on integrating results concerning miRNA expression from different body fluids to improve NSCLC detection (124). The participation of miR-145 has been repeatedly proven by scientists to be involved in NSCLC (63, 125, 126). An interesting and comprehensive systemic review and meta-analysis of that miRNA was published in 2020. The authors found nine studies (with 11 data sets) concentrated on miR-145 role in NSCLC, and as a result, the summary ROC of the miR-145 for the detection of NSCLC amounted to AUC = 0.83. Due to the authors, miR-145 can potentially be a diagnostic biomarker of NSCLC, but further studies are still required (60). Another group investigated serum miRNAs from the dataset GSE24709, which were: miR-432, miR-942, miR-29c-5p, and miR-601. Data validation was carried out on 20 NSCLC patients and 20 healthy volunteers allowing a selection of miR-942 and miR-601 as the most up-regulated in NSCLC patients. Further analysis on a larger cohort allowed the authors to confirm significantly higher levels of these miRNAs in NSCLC patients than in healthy controls. Furthermore, these miRNAs performed better than CEA, CYFRA21-1, and the SCC antigen in the early detection of NSCLC (61). One of the recent studies on Indian NSCLC patients aimed to validate serum miRNAs, which were selected as differentially expressed after small RNA sequencing. The authors found miR-15a-5p, miR-320a, miR-25-3p, miR-192-5p, miR-let-7d-5p, miR-let-7e-5p, miR-148a-3p, and miR-92a-3p dysregulated in serum of NSCLC patients. Additionally, miR-375 and miR-10b-5p were significantly down-regulated in SCC patients compared to controls. Let-7d-5p showed the highest diagnostic value with AUC = 0.917 and a sensitivity of 76% at 100% specificity (62). It is worth confirming these studies on larger groups of patients from other populations to assess the diagnostic value of the miRNAs included in this study. Microarray analysis indicated that the serum exosomal miR-20b-5p and miR-3187-5p were remarkably lower in NSCLC patients compared to healthy subjects. Next, it was verified via RT-qPCR on larger groups containing 276 NSCLC patients and 282 healthy controls. Combining both miRNAs with CEA or CYFRA21-1 showed AUC = 0.905 for miR-20b-5p and AUC = 0.894 for miR-3187-5p. Furthermore, integrating those two miRNAs could discriminate patients at an early stage from healthy controls (64). An undoubted advantage of this study was the size of the tested groups. Nevertheless, the authors point out the need to examine the potential utility of these miRNAs to distinguish NSCLC patients from patients with benign lung diseases. Several studies proved that the miR-17-92 gene cluster participates in cancer development by targeting mRNAs involved in distinct pathways that promote or inhibit carcinogenesis (127–130). This cluster consists of six miRNAs: miR-17, miR-18a, miR-19a, miR-19b-1, miR-20a, and miR-92a-1. The latest research by Yang et al. (65) aimed to examine the potential diagnostic utility of the miR-17-92 gene cluster in NSCLC. ROC analysis proved that the miR-17-92 gene cluster might be helpful as a diagnostic NSCLC marker. Furthermore, the high positive correlation between miR-17 and miR-20a expression was observed, indicating these miRNAs could be used together as a panel to improve the accuracy of the NSCLC diagnosis. Serum miR−518b was up-regulated in NSCLC serum, tumor tissues and cell lines (A549, H1299, H1975, and PC9) compared to coherent healthy controls. The diagnostic accuracy of miR-518b was supported by ROC analysis, where the AUC value was 0.910, with 88.1% sensitivity and 81.7% specificity (66). A recent study by Zhang and Xu (67) indicated that combining exosomal miR-378 with CEA could accurately differentiate NSCLC patients from healthy donors. The role of miR-185 in oral cancer (131), prostate cancer (132) and in NSCLC has been previously evaluated (133, 134). In 2020, Liu et al. (68) demonstrated that serum miR-185 might be a promising biomarker for NSCLC early detection. Their work showed significantly reduced miR-185 expression in NSCLC patients with lymph node metastasis at the advanced stage or with poor differentiation. Moreover, serum miR-185 demonstrated better diagnostic accuracy than CEA for distinguishing patients with carcinoma from disease-free controls. Xue et al. (69) identified 12 miRNAs profiling studies and analyzed differentially expressed miRNAs according to GEO2R online tool and RRA method from R. Performing validation via RT-qPCR revealed reduced miR-1228-3p serum levels (P = 0.006) and increased miR-181a-5p serum levels (p = 0.030) compared to healthy controls. ROC analysis results indicate the potential utility of miR-1228-3p and miR-191a-5p as NSCLC diagnostic biomarkers. Wang et al. selected four miRNAs (miR-1269a, miR-205-5p, miR-210-5p, and miR-9-3p) from TCGA database, which occur abundantly in serum exosomes from NSCLC patients. Their further analysis proved that these miRNAs may serve as novel diagnostic panel distinguishing NSCLC patients from healthy subjects (71). Another authors found lower miR-186 levels in serum and exhaled breath condensate (EBC) of NSCLC patients compared to healthy donors. The analysis showed that the SCC group had a higher serum interleukin-1β level than the ADC. Moreover, a larger AUC was obtained when the interleukin-1β and miR-186 levels were combined (72). Yang et al. (74) indicated the potential diagnostic role of exosomal miR-21/Let-7a ratio, which was up-regulated in NSCLC patients compared to healthy controls, patients with pulmonary inflammation diseases, and benign pulmonary nodules. Analysis revealed that combined levels of both miRNAs could precisely differentiate NSCLC patients from pulmonary nodules diseases and healthy controls. This study highlights that combining more than one miRNA may have better diagnostic value than testing individual miRNAs’ alone. A limitation of this study was the relatively small number of patients in the groups. However, it has the advantage of comparing NSCLC (n = 75) not only with healthy controls (n = 24) but also with benign pulmonary nodules (n = 23) and pulmonary inflammation diseases (n = 18). Analysis of larger patient cohorts is needed to more precisely assess the value of exosomal miR-21/Let-7a ratio to serve as NSCLC biomarker (74). Wu et al. (76) conducted a groundbreaking study exploring the same eight serum and exosome-derived miRNAs. Significant miRNA up-regulation was observed in four early-stage NSCLC serum particles (miR-21-5p, miR-141-3p, miR-222-3p, and miR-486-5p) and two exosomal ones (miR-146a-5p and miR-486-5p) compared to patients with benign lung lesions and healthy donors. Combining the results of over-expression of these miRNAs gave the best results in terms of the diagnostic power of the test. (AUC = 0.960, with a sensitivity of 85.42% and a specificity of 92.50%). These findings encourage an exploration of the potential utility of combined serum and exosomal miRNAs, which can improve NSCLC early diagnosis. Results from the study conducted by Ding et al. (77), indicated serum miR-184 down-regulation and miR-191 up-regulation in NSCLC patients compared to controls and pneumonia patients. Further analysis proved that these miRNAs may potentially serve as diagnostic biomarkers in NSCLC. Performing a ROC curve analysis showed better diagnostic efficacy when both miRNAs were combined. In turn, levels of miR-93-5p and miR-18a were significantly up-regulated in NSCLC patients compared to disease-free controls. The AUC value for both mRNAs in NSCLC diagnosis was high (0.89). According to the authors, further analysis of larger cohorts is needed to understand the role of these miRNAs in NSCLC (79). Another group recognized miR-891a-5p to have diagnostic value in NSCLC. Increased levels of miR-891a-5p were notably increased in serum, tissue and NSCLC cell lines (H1299, HCC827, H460, and A549). Expression levels of serum miR-891a-5p could accurately distinguish NSCLC patients from the healthy controls with the AUC = 0.904 (81). The study of Kryczka et al. (82) in 2021 was focused on finding diagnostic NSCLC biomarkers in serum exosomes. Among four surveyed miRNAs, the authors found miR-23a and miR-let7i significantly down-regulated in NSCLC compared to healthy controls. All vehicle-derived miRNA included in the research (miR-23a, miR-361, miR-1228 and miR-let7i) were connected in panels, and results showed better diagnostic values with AUC of 0.705 for miR-23a and miR-let7. Zhao et al. (88) indicated that serum miR-205-5p could accurately differentiate patients in early NSCLC stage (I and II) and advanced stage (III and IV) when compared to healthy controls, with the AUC of 0.8141 and 0.8045, respectively. However, some studies on miR-miR-205-5p have been contradictory, so there is a need to precisely define the role and utility of this miRNA in diagnosing NSCLC (135). MiR-138-5p was found under-expressed in NSCLC cell lines (A549, H1975, and PC9), serum and lung ADC tissues. NSCLC could be distinguished accurately from healthy controls by miR-138-5p with an AUC of 0.922, which supports this miRNA potential utility in NSCLC diagnosing (90). In another study, microarray analysis of miRNA from four NSCLC patients and five healthy subjects helped to select miR-1247-5p, miR-301b-3p, and miR-105-5p. Next, those miRNAs were validated via qPCR on 154 NSCLC patients and 146 controls. All those miRNAs were highly expressed in diseased patients’ plasma, and further analysis proved its role in NSCLC tumorigenesis. After combining those three miRNAs with CEA, ROC analysis showed higher sensitivity and specificity than CEA alone (97). A study conducted by Zheng Q et al. (99) suggests that exosomal miR-1246 and miR-96 from plasma may be helpful in diagnosing patients with NSCLC. However, miR-96 showed significantly higher diagnostic potential (AUC = 0.9735) than miR-1246 (AUC = 0.6761). Wu et al. (100) focused on human papillomavirus (HPV) infection, which seems to be a dangerous risk factor in cancer patients (136–138). They found that miR-183, miR-210, and miR-182 were significantly higher, and miR-144 was markedly lower in HPV-DNA-positive NSCLC than in HPV-DNA-negative NSCLC patients. Notably, miR-210 combined with miR-144 had the best prediction performance in diagnosing HPV-DNA-positive NSCLC patients. In the subsequent study from 2021, a reduction of plasma miR-216b was observed in early, advanced ADC and SCC patients. Lower levels of that miRNA were also observed in early ADC and SCC tissues compared to adjacent ones. Results of this study demonstrated that miR-216b reduced expression was correlated with the tumor staging, indicating this miRNA potential utility in NSCLC patients classification (101). Jiang et al. (103) selected 12 previously found dysregulated miRNAs in NSCLC, investigated them via RT−qPCR in the training group, and selected four miRNAs (miR−210, miR−1290, miR−150, and miR−21−5p), which were subsequently validated on the testing set. Expression of all four miRNAs proved to be over-expressed in NSCLC patients compared to patients with benign lung disease and healthy donors. Combining all tested miRNAs as a panel showed a higher diagnostic performance than individual miRNAs alone. The limitation of the study was too short follow-up and a relatively small number of included patients. A study by Jiang (108) aimed to investigate the role of plasma circulating small extracellular vesicles (sEVs) in early-stage NSCLC and SCLC. Next Generation Sequencing revealed six dysregulated miRNAs (miR-483-3p, miR-152-3p, miR-1277-5p, miR-130b-3p, miR-25-5p, and miR-4429), which were further validated via RT-qPCR. The results showed that sEVs plasma miRNA−152−3p and miRNA−1277−5p might be used to diagnose early−stage NSCLC patients. Wu et al. (109) found plasma miR-340 expression decreased and plasma miR-450b-5p increased in NSCLC patients compared to healthy donors. AUC values were 0.740 and 0.808, respectively. When both miRNAs were combined, the AUC value was 0.862 with 78.33% specificity and 77.5% sensitivity. It is worth conducting more detailed analyses to assess the value of both these plasma miRNAs as a panel. Vadla et al. (110) drew attention to the problem of false positive NSCLC diagnosis, which leads to unnecessary testing and treatment. The authors uncovered that combining plasma miR-22-3p, vesicle-derived miR−184, and miR-let−7b−5p distinguished NSCLC patients from high-risk subjects. This valuable research highlights the potential role of those miRNAs in improving the precision of NSCLC diagnosis. The validation of miR-34c-5p via qRT-PCR showed its up-regulation in NSCLC plasma samples from males compared to healthy donors (*p* = 0.004, AUC = 0.8467). These findings were consistent with the microarray results on tissues and plasma samples (111). In the newest meta-analysis by Wang et al. (139), it is concluded that miR-21 from serum or sputum can be a promising biomarker in the identification of patients with lung cancer, including NSCLC, with high precision. In this paper, four studies tested the miR‐21 expression in sputum and 13 in serum samples. In both cases, the specificity and sensitivity for miR-21 were relatively high.

Several studies have reported that NSCLC diagnosing can be aided by determining miRNAs in the patient’s sputum. Sputum is a kind of mucus that is an expectorated secretion of the respiratory system. It includes epithelial cells from the lungs and lower respiratory tract, microbial products, inflammatory cell components, and traces of saliva. It is readily available, easy to collect, cost-effective, and can reflect the conditions of the lungs (140–142). Sputum collection may be facilitated by inhalation of nebulized hypertonic saline solution (143).

In the meta-analysis combining results from 14 articles, the authors suggest that the integration of miR-210, miR-21, and miR-31 from sputum may be a good biomarker for NSCLC diagnosis (144). The next meta-analysis study shows the high diagnostic significance of miR-210 in the serum and sputum of NSCLC patients. The authors analyzed data from GEO and TCGA. Indicated AUC values for this miRNA were 0.82 in the serum and 0.81 in the sputum (145). A recent study by Su et al. (114) revealed that the combination of miRNA with other non-coding RNAs might be profitable in the diagnosis of NSCLC patients. Integrating three sputum miRNAs (miR-21, miR-32, and miR-210) and two sputum small nucleolar RNAs (SNORD66, SNORD78) synergistically affect lung cancer detection. It showed higher sensitivity and specificity than miRNA or those small nucleolar RNAs analyzed alone. In 2018 (115), the same authors integrated two sputum miRNAs (miR-31 and miR-210) and miR-19b-3p from peripheral blood mononucleated cells (PBMC) in the NSCLC group. This panel had higher sensitivity and specificity than these miRNAs analyzed separately. The above studies were the continuation of previous research by these scientists, which have proven the potential utility of sputum miR-21, miR-31, and miR-210 as NSCLC biomarkers (120, 146). The logistic regression model based on miR‐145, miR‐126, and miR‐7 expression, obtained by qRT-PCR reaction, showed 0.93 AUC in the lung cancer group, which is a promising result for NSCLC diagnosis. Furthermore, the miRNA showed stability in the sputum even after one year of storage. The main limitation of this study was the relatively small specimen number, which consisted of 30 NSCLC patients and 30 healthy controls (116). Li et al. (119) indicated using digital droplet PCR, a combination of biomarkers sputum miR-31-5p and miR-210-3p, sputum DNA methylation *RASSF1A*, and two plasma miR-21-5p and 126 in NSCLC diagnosis. The integration of these three components showed AUC = 0.956, which is higher than a single type of biomarker. The results were confirmed in a validation cohort, which indicated the high potential of this combination in NSCLC diagnosis. Another study of these authors showed that combining sputum miR‐31‐5p and miR-210‐3p with plasma miR‐21‐5p synergistically affected early NSCLC diagnosis (117).

Sputum appears to be a very promising source of miRNAs diagnosing NSCLC. However, the number of publications focusing on miRNAs in sputum is limited compared to those conducted on serum and plasma. We have not found any studies demonstrating a clear prognostic and predictive potential of sputum miRNAs in NSCLC. The studies presented here require further validation and testing in more significant numbers of patients. It is worth noting that miR-21, miR-31, and miR-210, in particular, are repeated in several studies demonstrating their efficacy in diagnosing patients with NSCLC. However, most of these studies were conducted by the same authors, so the groups of patients may have come from a similar population. Independent research centres should confirm these results on independent patient groups. Analyzing the studies, the conclusion is that miRNAs assembled in panels have more significant diagnostic potential than those miRNAs analyzed separately. This usually increases the accuracy and precision of the diagnostic test.

## 3 Circulating miRNAs as prognostic biomarkers for NSCLC

TNM Classification of Malignant Tumors plays a significant role in NSCLC prediction and prognosis, allowing for the estimation of patient performance status, survival time, histologic tumour grade, and relapse rate. For instance, the presence of any malignant pleural effusion, distant disease or contralateral nodule is associated with 5-year survival rates of less than 6%. However, searching for new molecular and clinical biomarkers is essential since heterogeneity in outcomes is perceived among the same TNM groups. Identifying prognostic biomarkers for NSCLC is of great importance to refine risk stratification and guide treatment planning (147, 148).

In 2020 (53), serum and tissue miR-30 levels were found down-regulated in NSCLC patients compared to healthy donors. Importantly, a statistically significant difference was found in the median overall survival (OS) between patients with under-expression of miR-30 (23.0 months) and patients with over-expression of that miRNA (36.0 months). Levels of miR-30 were correlated with lymph node metastasis, tumor size, tumor node metastasis (TNM) stage, and differentiation degree. Huang and Qu (54) demonstrated that the patients with higher levels of serum exosomal miR-1246 had poorer OS and disease-free survival. Further analysis showed that the level of that miRNA, as well as, the TNM stage and lymph node metastasis, were proven to be independent prognostic factors in NSCLC. Another study aimed to determine whether circulating serum miR-762 can improve the NSCLC diagnosis and prognosis. The results showed up-regulation of this miRNA in NSCLC patients with the advanced clinical stage (III and IV), positive lymph node metastasis and poorly differentiated tumors towards patients at the early clinical stage. Furthermore, it has been observed that the level of miR-762 was statistically associated with histological grade, clinical stage, and lymph node metastasis (56). It has been shown that miR-519b expression in NSCLC tissue and serum samples was markedly lower. The association between lymph node metastasis and clinical stage have been observed, as well as significantly shorter OS rates than patients with higher serum miR-519d levels. Notably, the cell-based part of the research has revealed that miR-519d regulates NSCLC progression by targeting human epidermal growth factor receptor 3 (57). Zhou et al. (61) indicated serum miR-942 and miR-601 as over-expressed in NSCLC patients compared to control. Their further analysis showed the potential role of both miRNAS as NSCLC prognostic biomarkers. Higher levels of one tested miRNA (miR-942 or miR-601) were connected with adverse clinical variables and poor survival of NSCLC patients. Interestingly, the most favourable outcome could be observed in NSCLC patients with low levels of miR-942 and miR-601. A recent study conducted on Indian NSCLC patients (62) pointed to a potential prognostic role of serum miR-375 and miR-10b-5p. Levels of that miRNAs were down-regulated in SCC patients compared to controls. Authors observed significant correlation of miR-375 with lymph node involvement (p = 0.0224) and with pleural effusion (p = 0.0148), while miR-10b-5p association was seen only with the pleural effusion (p = 0.0037). In 2020, X. Zhang et al. (66) found increased levels of serum miR-518b, which was observed also in tissues and cell lines compared to corresponding biological material. Over-expression of miR-518b was significantly associated with tumor size (*p* = 0.042), advanced TNM stage (*p* = 0.006) and lymph node metastasis (*p* = 0.039). Significantly, miR-518b over-expression was likewise associated with shorter survival of patients (*p* = 0.009). J. Liu et al. (68) as first demonstrated that decreased level of serum miR-185 was markedly associated with worse survival and adverse clinicopathological parameters in NSCLC patients. Performing univariate and multivariate Cox regression analysis indicated that this miRNA was an independent prognostic indicator for NSCLC. Another study investigated the levels of serum miR-1228-3p and miR-181a-5p in 50 NSCLC patients. They showed that increased levels of miR-1228-3p and decreased levels of miR-181a-5p were significantly associated with OS. MiR-1228-3p was characterized as independent factor for poor prognosis in Cox regression analysis (69). In the study by Kumar et al. (70), miR-3195 from serum was characterized as an independent prognostic factor for OS. Patients with higher levels of that miRNAs are predisposed to significantly longer OS (p = 0.0298). In a study conducted by Xu et al. (75), serum exosomal let-7e could differentiate NSCLC patients from healthy controls. This tendency has also been noticed in tissues. Kaplan–Meier method showed that miR-let-7e expression and higher *SUV39H2* expression were correlated with lower survival rates of NSCLC patients. Shao et al. reported a significant increase in levels of miR-93-5p and miR-18a in 107 NSCLC patients compared to osimertinib controls (n = 42). Both miRNAs’ expressions were associated with tumor differentiation degree, TNM stage, lymph node metastasis and lymph-vascular space invasion. Another study was focused on exosomal miR-382, which turned out to be significantly down-regulated in NSCLC patients. Interestingly, further research revealed that most of the patients, after surgical resection, had increased miR-382 expression (80). The results of another study showed up-regulated expression of miR-891a-5p in serum and tissues from NSCLC patients compared to NSCLC cell lines. According to the authors, miR-891a-5p might serve as a prognostic NSCLC biomarker, inferred from association with differentiation, tumour, node and metastases stages (81). Serum miR-629 was another example of up-regulated miRNA in NSCLC compared to controls. Dysregulation of that miRNA was positively related with lymph node metastasis, differentiation and clinical stage. Moreover, patients with higher miR-629 levels had poorer OS and disease-free survival than subjects with lower levels of miR-629 (84). Wang et al. found that miR-192 and miR-194 were remarkably lower in NSCLC patients. Both miRNA levels were linked to the TNM, distant metastases, and pathological stages. Moreover, miR-192 was as well correlated with the pathological stage (85). In another study from 2021 (88), serum levels of miR-205-5p were found to increase and it was associated with patients’ gender, drinking status, and clinical stage. In turn, exosomal miR−433 was down-regulated in chemotherapy-resistant NSCLC plasma patients compared with chemotherapy-sensitive NSCLC and normal serum. Expression of this miRNA was negatively associated with large tumour size, distant metastasis, advanced TNM stage and poor prognosis (89). The research of Zeng et al. (91) showed that the expression miR-31-3p level was significantly higher in serum and NSCLC tissue. Further analysis showed that high expression of miR-31-3p with the TNM classification, and lymphatic metastasis could be used as risk factors and independent predictors of bone metastasis, while tumour size could be used as a risk factor for bone metastasis. The role of miR-30a-5p in ADC was raised in a recent study by Jiang et al. (92). According to the results of their work, expression of that miRNA was significantly decreased in ADC cell lines, tissues and patients’ serum. Lower levels of miR-30a-5p were, inter alia, linked with the TNM stage, pathologic stage, gender, and smoking (92). Another study showed that plasma miR-590-5p levels were dramatically down-regulated in NSCLC patients compared to healthy controls. Importantly, patients with stages III and IV had marginal down-regulation of that miRNA compared to healthy controls. Furthermore, Kaplan-Meier and log-rank analyses revealed a negative correlation of miR-590-5p in the prognosis of NSCLC patients (93). The recent Khandelwal et al. (102) study detected that plasma miR-320a was under-expressed in NSCLC patients. Furthermore, levels of that miRNA were associated with tumor size, TNM stage, and lymph node metastasis. In another study, the authors highlighted the problem of bone metastasis in NSCLC patients. The purpose of their research was to find high-accuracy biomarkers to be alternatives for skeletal scintigraphy, computerized tomography (CT), positron emission tomography-computed tomography (PET-CT), and magnetic resonance imaging (MRI). Within one of the tested miRNA clusters, the authors found significant under-expression of plasma-derived exosomal miR-574-5p, over-expression of plasma-derived miR-328-3p and miR-423-3p in patients with bone metastasis compared with subjects without that ailment (stage IV) (104). Jiang et al. (103) investigated the role of four plasma miRNAs (miR-210, miR-1290, miR-150, and miR-21-5p) in NSCLC prognosis and diagnosis. Kaplan−Meier survival analysis demonstrated that two miRNAs (miR-210 and miR-150) were significantly correlated with a shorter disease-free survival time in patients without SCC lung cancer, but not in SCC patients. Significant differences were not observed in plasma levels of miR−210 and miR−150 between patients with SCC and patients without SCC lung cancer. Kim et al. (106) analyzed plasma exosomal miR-1260b role in NSCLC. Their results showed its up-regulation in tissues, plasma and human umbilical vein endothelial cells. These levels were connected with high-grade disease, metastasis, and poor survival. Another study characterized plasma miR-340 and miR-450b-5p as independent biomarkers of survival in non-metastatic NSCLC patients. Besides, plasma miR-340 was also negatively correlated with tumor grade (109).

## 4 Circulating miRNAs as predictors of NSCLC treatment response

Drug- and radio-resistance in NSCLC patients are major causes of therapeutic failure. Hence, choosing the right way of treatment is crucial to prevent tumor recurrence, disease progression and unnecessary side effects. Some authors indicate that routine treatment with EGFR-TKI should be avoided in molecularly unstudied populations (149, 150). Accumulating evidence has shown that miRNA concentrations might be varied between treatment-sensitive and treatment-resistant NSCLC patients. This paragraph presents recent studies focused on the search for miRNAs, which have predictive potential in NSCLC treatment response.

Tang et al. (55) revealed the potential role of exosomal-derived serum miR-620 as a predictive biomarker in NSCLC patients. Results showed an association of lower levels of that miRNA with chemotherapeutic effect (*p* = 0.044), which can be helpful in the prediction of response to chemotherapy. Moreover, significant down-regulation of that miRNA was observed in patients with metastatic NSCLC, showing its potential in the prediction of future metastasis. According to the research of Zhang et al. (58), exosomal miR-5684 and miR-125b-5p might serve as prognostic biomarkers in NSCLC. Under-expression of these miRNAs was observed in diseased serum samples compared to healthy controls. Importantly, levels of exosomal miR-5684 and miR-125b-5p were significantly associated with metastasis (p < 0.0001), chemotherapeutic effect (P = 0.007) and survival (P = 0.008). This study was characterized by a relatively large study group what makes the results convincing. However, the authors emphasize the importance of further examining the insights mechanism of exosomal miR-5684 and miR-125b-5p. A recent study by Zhang and Xu (67) reported that the serum exosomal miR-378 was notably up-regulated in NSCLC patients. These levels were significantly related to positive lymph node metastasis and advanced TNM stage. Among the many interesting observations, the level of miR-378 was decreased after radiotherapy and could be potentially used as an indicator of radiotherapeutic response. Kumar et al. (70) as first investigated the role of miR-3692-3p, miR-3195, and miR-1249-3p as circulating NSCLC biomarkers. The results indicated the potential usefulness of miR-1249-3p in prediction of therapeutic response to chemotherapy. Moreover, it was found that levels of miR-1249-3p were significantly higher in ADC versus SCC (*p* = 0.0178). The subsequent interesting study has indicated serum-derived miR−130a, miR−25, and miR−191 up-regulation in NSCLC patients compared to disease-free controls. These levels were associated with advanced age (≥ 60 years), radiotherapy, histological type (SCC), low survival rate and low median survival time. These miRNAs could potentially serve as predictors of radiotherapy outcome in NSCLC patients (73). Gefitinib is the first selective inhibitor of epidermal growth factor receptor’s (EGFR) tyrosine kinase domain that was the first targeted drug that entered clinical practice for treating lung cancer (151). Ding et al. point to potential usefulness of serum miR-184 and miR-191 as biomarkers for predicting gefitinib efficacy in NSCLC patients. Results showed that increased miR-191 and decreased miR-184 expressions in NSCLC patients were associated with a higher risk of ineffective gefitinib treatment (77). Chen et al. (83) also indicated the potential prognostic potential role of miRNAs in treatment predicting by gefitinib. Exosomal miR-7 from serum was found dysregulated and it was associated with a longer survival rate of patients, early tumor size and a solid reaction to gefitinib treatment. Osimertinib is the first third-generation epidermal growth factor receptor (EGFR) tyrosine kinase inhibitor (TKI) approved for treating locally advanced or metastatic NSCLC in patients with epidermal growth factor receptor (EGFR T790M) resistance mutation (152). Li et al. (86) found up-regulation of exosomal miR-184 and miR-3913-5p in NSCLC patients after the onset of osimertinib resistance. MiR-184 amounts were correlated with lactate dehydrogenase levels, whether expression of miR-3913-5p with TNM stage, platelet count, CEA and metastases. This study suggests that these two miRNAs may serve as biomarkers for detecting NSCLC patients being resistant to osimertinib. In another study, serum exosomal miR-1169 and miR-260 have been presented as potential biomarkers discriminating between wild-type epidermal growth factor receptor (EGFR) and mutant EGFR NSCLC at an early stage (87). In turn, serum exosomal miR−433 was down-regulated in chemotherapy-resistant NSCLC patients’ plasma compared with sensitive NSCLC and serum of healthy donors. Expression of this miRNA was negatively associated with large tumor size, distant metastasis, advanced TNM stage and poor prognosis. Platinum-based chemotherapeutics, mainly cisplatin and carboplatin, are routinely used for the treatment of lung cancer (153, 154). Plasma exosomal miR-1273a was found significantly under-expressed in the non-responder NSCLC patients after cisplatin therapy. Furthermore, a significant association was observed between miR-1273a low levels and worse therapeutic outcomes of advanced NSCLC subjects receiving platinum-based chemotherapy. This study demonstrates that miR-1273a is related to cisplatin resistance and might be helpful in NSCLC prognosis and therapy (94). Another research by Peng et al. (95) revealed higher levels of plasma miR-320d, miR-320c, and miR-320b in the progressive NSCLC disease group compared with the achieved partial response group at the beginning of the therapy. Furthermore, decreased plasma exosomal miR-125b-5p levels were observed in post-treatment patients, which might help to gain greater T-cell function and respond well to immunotherapy. Papadaki et al. (96) researched miRNAs (plasma miR-21, miR-128, miR-155, and miR-181a) which involvement in damage response (155) and tumor responsiveness to platinum was previously studied (156, 157). According to the results, these miRNAs were significantly up-regulated in NSCLC patients compared to healthy controls. Among interesting findings, miR-128 was associated with worse OS, whereas miR-155 with OS in SCC with platinum-based chemotherapy. Those two miRNAs may function as independent predictors in platinum-based chemotherapy. Leonetti et al. (98) conducted a comprehensive study to explore the role of plasma miRNAs (miR-21, miR-27a, and miR-181a) which could potentially serve as substitute for EGFR-TKI treatment. The level of miR-21 was observed as up-regulated in NSCLC patients who partially/completely responded to the treatment, when compared to patients with disease stability/progression. Moreover, sixth-month lasting clinical benefits patients have shown higher basal levels of circulating miR-21 (*p* = 0.039). Another study aimed to examine the possible utility of sixth exosomal miRNAs (miR-21, miR-1246, miR-let-7g, miR-210, miR-214, and miR-96) from plasma, but only miR-1246 and miR-96 were significantly up-regulated in NSCLC patients. Interestingly, exosomal miR-96 in patients with radioresistant NSCLC was observed significantly up-regulated and was correlated with poor prognosis (99). Another study by Zuo et al. (101) showed down-regulation of plasma miR-216b in early and advanced SCC patients, what corresponded to the tissue results. Moreover, plasma miR-216b was negatively correlated with 18F-fluorodeoxyglucose (18F-FDG) uptake in NSCLC. According to the results, 18F-FDG could potentially serve as a predictor of therapeutic response in the application of miR-216b-based cancer treatment. In turn, up-regulation of plasma miR-202 and miR-26a was found in advanced NSCLC patients treated with platinum-based chemotherapy. High level of miR-202 was associated with disease progression and was shown as an independent prognostic factor for shorter progression-free and OS. Whilst miR-26a was correlated with shorter OS in SCC (105). Another study presented the role of plasma miR-200c and miR-34a advanced NSCLC treated with anti-PD1 immunotherapy. Over-expression of miR-200c was characterized as an independent prognostic factor for inferior OS in all NSCLC and most non-SCC patients. In turn, the low miR-34a level was connected with shorter OS in non-SCC (107). Marconi et al. first showed that combining exosomal plasma miRNA with peptidome might serve as an NSCLC biomarker to identify patients with a higher risk of recurrence after surgery. They investigated the role of plasma exosomal miR-130a-3p and fibrinopeptide A (FpA), and a significant correlation with DFS was observed in NSCLC patients. According to the authors, these findings may help predict early-stage NSCLC patients predisposed to relapse after surgery (112). Pantano et al. (113) conducted a large-scale profiling of plasma extracellular miRNA vesicles. They characterized plasma EV-miR-625-5p as an independent biomarker of response and survival in NSCLC patients treated with ICIs, particularly those with the programmed death ligand -1 expression ≥ 50%.

## 5 The challenges of circulating miRNAs as cancer biomarkers

The discovery of miRNA molecules allowed science and medicine to expand the knowledge about their functions and mechanisms of action in the human body. MiRNAs were found to be differentially expressed in various tissue and cell types, which indicated their participation in many essential processes, such as proliferation or carcinogenesis (158). Many studies have indicated their potential utility as diseases biomarker, including cancer, but none of the miRNAs studied so far has been successfully introduced into routine clinical practice. A biomarker, by definition, is “objectively measured and evaluated as an indicator of normal biological processes, pathogenic processes, or pharmacologic responses to a therapeutic intervention”. Moreover, biomarkers should be non-invasive, easy to measure, detectable from multiple sources, and have high specificity, allowing for early diagnosis (159).

MiRNAs have many features of a promising biomarker, so extensive research into their potential utility in this area is well-funded. Unfortunately, many studies reveal contradictory results which may be caused i.a. by the lack of standardized procedures. They consist of the pre-analytical phase, including sample collection and preparation; the analytical phase, which determines the miRNA expression levels; and the post-analytical phase, handling data extraction and normalization (160). Collecting of material for research is a challenge, even for typical materials like serum and plasma. The concentration of serum miRNAs in higher than in plasma, which may indicate that the coagulation process may affect the change of the miRNA profile (161). The limitation in using plasma as a tested material is adding the heparin, which may cause inhibition of the Taq polymerase during the amplification (162).

The selection of patient groups also has an undoubted influence on the results. Apart from the disease state, miRNA expression can be affected by many other factors, such as diet, physical activity, and chemotherapy treatments. Many of these changes are difficult to assess, so it is essential to standardize the groups and the results should be confirmed on large independent cohorts. If several studies on the same miRNAs come from similar populations, selected groups may not have been sufficiently diverse (163). Dysregulation of miRNAs can often result not only from a disease state but also from the activity of the immune system (164). It could be the explanation for why the altered expression of the same miRNAs is observed in many studies. An important aspect is selecting an appropriate large group to obtain sufficient study power. The possibility of getting random results can be reduced by eliminating bias in the study design using techniques such as randomization and blinding (165).

Many methods and kits are used to isolate miRNAs, which makes comparing results less reliable. Moreover, miRNA detection can be affected by inhibitors of the qPCR, which may be present after isolation procedure (166). Mestdagh et al. compared nine different platforms for measuring serum miRNA expression. The high accuracy with a strong sensitivity was observed in the qPCR method. These findings indicate the need to confirm the results with at least two methods (167).

Despite its many limitations, miRNA has considerable potential to become a biomarker for many diseases, including NSCLC. Above all, developing standard operating procedures at each stage of miRNA processing can minimize the disadvantages of using miRNA as a biomarker.

## 6 Concluding remarks

The latest studies searching for biomarkers for NSCLC in body fluids have focused mainly on serum and plasma. A highly interesting and still not very popular direction in the search for lung cancer biomarkers is evaluating the expression of miRNAs isolated from patients’ sputum. However, other biofluids, such as urine and saliva, should be considered in future research. We found only one study which proved that urine exosomal miR-4466 levels in lung cancer patients could be a biomarker for predicting an increased risk of metastatic disease among smokers (168). Interestingly, many of the studies we have cited revealed that miRNA levels are not significantly related to age, gender, tumour characteristics and tumour size (56, 66, 86, 115, 145). These observations suggest the potential usefulness of miRNAs in screening, regardless of the patient’s age or gender. It still needs to be determined, which biofluid provides the best chance of finding accurate NSCLC biomarker, especially the research results are inconsistent.

MiRNAs play a significant role in many processes leading to the development of NSCLC and its consequences. The studies above proved that these particles have many features of an ideal potential biomarker for molecular diagnosis, prognosis, and monitoring of NSCLC. Their use provides a promising alternative to other diagnostic methods, which are invasive and often imprecise. Nevertheless, there are also some limitations in miRNAs reliability for practical clinical application. For instance, dysregulated levels of the same miRNAs are observed in different types of cancer (169).

Among the research papers described in this review, we selected miRNAs with the highest diagnostic value and presented them in **Table 4**. Several studies indicate that miRNAs combined into panels or combined with an additional parameter, such as CEA and CYFRA21-1, had a higher diagnostic value than these biomarkers analyzed separately. These miRNAs and panels are worth considering in future studies to improve the diagnosis of NSCLC. Unfortunately, many studies did not have a validation group, and the authors only used an unbiased screen and separate discovery and validation cohorts in three studies. Most of authors used qPCR/qRT-PCR in their studies, which is a valid approach for testing miRNAs. However, researchers should also confirm their findings with another available method (167).

Our review suggest that miRNA extracted through the use of liquid biopsy appears to be an ideal biomarker for the diagnosis, prognosis and prediction of NSCLC. However, researchers should be aware of the numerous challenges of using miRNAs in these applications. Scientists should aim to develop standard operating procedures for material collection, storage, choice of detection platform, analysis of miRNA profile, and data normalization (166, 170). All of those factors may have an influence to the levels of circulating miRNAs. Moreover, levels of miRNAs may depend on age, gender and previous therapies used by patients, causing misinterpretation of the results (171). The most sensitive and high-throughput miRNA examing methods, such as Next Generation Sequencing, digital PCR and Nanostring Technology, prove to be invaluable in dispelling many doubts about the usefulness of some miRNAs as NSCLC and other disease biomarkers. To avoid false positive results, it is worth finding miRNAs that differ as much as possible in the expression level between NSCLC patients and healthy individuals (172).

Researchers should constantly strive to standardize and normalize the sampling, storage, isolation and subsequent detection of miRNAs to avoid misleading findings. Therefore, there is a need to validate all promising results on larger groups of patients before miRNAs begin to be used in diagnostic and clinical practice. The new direction of current and recent research on miRNAs in the context of NSCLC and other cancers is an important target for future research.

## Author contributions

Conceptualization, JR, MN, AB and AK; supervision, MN and AK; writing—original draft preparation, JR; writing—review and editing, AB, MN and AK. All authors contributed to the article and approved the submitted version.
